# The deceptive simplicity of mendelian genetics

**DOI:** 10.1371/journal.pbio.3001691

**Published:** 2022-07-19

**Authors:** Aoife McLysaght

**Affiliations:** Smurfit Institute of Genetics, Trinity College Dublin, Dublin, Ireland

## Abstract

Gregor Mendel meticulously uncovered the genetic basis of heredity in work that transformed the science of biology. But does the alluring simplicity of Mendel’s laws sometimes obscure the true complexity of genetics?

## Teaser

Mendel, a genius experimentalist, meticulously uncovered the genetic basis of heredity in work that transformed the science of biology. But does the alluring simplicity of Mendel’s laws sometimes obscure the true complexity of genetics?

Gregor Johann Mendel was born 200 years ago this July. He is commonly referred to as the “father of genetics” because he was the first to understand that heredity works through the inheritance of discrete factors that come in pairs—what we now know as genes. This was a pressing question of the time, and had confounded no less a visionary than Charles Darwin, for whom it was the missing piece in understanding the mechanism of natural selection. Mendel’s expertly designed experiments, combined with his brilliant capacity for deduction, revealed the basis of heredity and thus the common language of all biology. To this day, his work is taught to every biology student; his name is invoked to describe certain kinds of inheritance; and, one might argue, the perceived simplicity of his results has sustained an unhelpfully simplistic view of genetics.

Though the vocabulary had not yet been invented, Mendel’s experiments comprising statistical analysis of meticulous crosses of pea plants laid the foundation for our understanding of the gene and the relationship and difference between inherited genes (genotype) and the physical form of an organism (phenotype) [1]. The idea was ahead of its time—35 years ahead of its time to be precise—but when his work was “rediscovered” in 1900, it finally found a receptive audience. This moment marked the beginning of an extraordinary period of discovery. William Bateson, who became a fervent champion of Mendel and coined the word “genetics,” correctly predicted that the science of heredity would be soon transformed [2,3].

Reading Mendel’s *Versuche* (“Experiments”) [1] today, it is striking to recognize our modern understanding of heredity in his results and interpretations, and it is easy to forget how far outside the contemporary mainstream ideas his conclusions were. Indeed, the conventional view that persisted into the early 20th century was that heredity occurred by blending of parental characteristics, a bit like mixing paint colors—a view shared by Darwin and his biometrician cousin Galton, who called it “Ancestral Heredity” [3,4]. The blending idea appeared, at least superficially, to fit quantitative traits such as human height, that we now understand to be complex genetic traits controlled by many individual genes, each with various forms (alleles or genetic variants) all inherited in a mendelian fashion.

In *Versuche*, Mendel devotes some time to explaining his experimental design [1]: He was careful to choose pea varieties that allowed a “sharp and certain separation,” and not ones where there was a gradient of forms. As distinct from complex genetic traits, the variation in these “mendelian” traits was largely controlled by a single gene. In fact, it is hard to imagine how anyone might have arrived at an accurate understanding of the mechanism of heredity from first principles by examining complex genetic traits—the multitude of different factors would seem to make it impenetrably complicated without an existing understanding of the nature of the gene. While we can readily understand complex genetic traits using a mendelian framework [5,6], a focus on complex traits may have been what prevented others, including Darwin, from understanding heredity [3,4]. The essence of experimental design is to simplify the problem just the right amount. Mendel was a genius experimentalist, with his work surely being the most advanced experimental biology of his time. He reduced the problem of heredity to one of beguiling simplicity.

I could not imagine trying to teach genetics without starting with Mendel. Genetics is incredibly (beautifully, fascinatingly, bewilderingly) complex. We now know that most traits—physical, biochemical, behavioral—are influenced by many different genetic variants, individually of small effect, acting in combination with the environment and stochastic processes during development. Even identical twins, who share all their genes, are not actually identical. And in terms of evolutionary genetics, we know that natural selection is most often acting on infinitesimal differences that only over long periods of time create eventual dramatic changes. But to explain any of this, it helps to first understand Mendel’s work and the simple experimental crosses where two versions of a gene generate forms that are sharply and certainly different, so we start there.

The important thing though is not to stop there. All too often, the popular conception of genetics is expressed with a phrase that begins “the gene for…” It is easy to see how the simplified scenario that Mendel necessarily constructed for his experiments could be misunderstood as the whole picture. It would appear to be irresistible to describe “the gene for eye color” or “the gene for tongue-rolling,” even though these classic, textbook examples of “mendelian” traits have been known to be multifactorial for decades. This temptation then spills over into discussion of “the gene for” any particular human trait or disease, when the reality is more often better described as a tendency or a propensity, which may or may not be realized. The effect of a genetic variant depends, to a greater or lesser extent, on the genetic and environmental context it happens to be in. Contrary to popular belief, the genes do not determine the trait, rather they shape the landscape of probabilities.

This deterministic view of genetics is the most insidious misconception of genetics—it is easy to learn and very difficult to abandon. It is culturally encoded in how we talk about inheritance, and becomes a hurdle to understanding and appreciating the powerful science of genetics [7]. In thinking genes determine everything, we risk losing sight of the true complexity of genetics and the intimate role of context. Someone carrying a genetic risk factor for, say, type 2 diabetes or heart disease, may never develop those conditions, and can even shift the balance of probabilities with healthy diet and exercise. And while a small minority of human diseases, including Huntington’s chorea and cystic fibrosis, are determined by variation at a single locus, even “simple” traits are often modified by more than one gene [6].

We are not mere vessels for our genes. Humans, uniquely, and starting with Mendel, are the only species that has developed an understanding of heredity and how genetic information is transmitted across generations and how genes help shape all biological life on this planet. Though we describe supposed single-gene deterministic traits as “mendelian,” I know of no evidence that suggests Mendel himself conceived of such a fanciful system. We honor Mendel by remembering his work, acknowledging its significance, and not attributing to him our modern-day errors of interpretation and oversimplification.
